# Instrumented Timed Up and Go Test as a Tool to Early Detection of Gait and Functional Mobility Impairments in Multiple Sclerosis

**DOI:** 10.3390/jcm15020679

**Published:** 2026-01-14

**Authors:** Piotr Szaflik, Aleksandra Kaczmarczyk, Hanna Zadoń, Justyna Szefler-Derela, Dagmara Wasiuk-Zowada, Katarzyna Nowakowska-Lipiec, Robert Michnik, Joanna Siuda

**Affiliations:** 1 Department of Biomechatronics, Faculty of Biomedical Engineering, Silesian University of Technology, Roosevelta 40, 41-800 Zabrze, Poland; katarzyna.nowakowska-lipiec@polsl.pl (K.N.-L.); robert.michnik@polsl.pl (R.M.); 2 Department of Neurology, Faculty of Medical Sciences in Katowice, Medical University of Silesia, 40-055 Katowice, Poland; akaczmarczyk@sum.edu.pl (A.K.); jsiuda@sum.edu.pl (J.S.); 3 Department of Clinical Engineering, Academy of Silesia, 40-555 Katowice, Poland; hanna.zadon@akademiaslaska.pl; 4 Department of Physiotherapy, Faculty of Health Sciences in Katowice, Medical University of Silesia, 40-754 Katowice, Poland; jszefler@sum.edu.pl (J.S.-D.); dwasiuk@sum.edu.pl (D.W.-Z.)

**Keywords:** multiple sclerosis, Timed Up and Go, TUG, instrumented Timed Up and Go, iTUG, gait sensors, motion analysis, spatial-temporal variables

## Abstract

**Background/Objectives**: Multiple sclerosis (MS) is a chronic demyelinating disease of the central nervous system that typically affects adults aged 20–50. Its early stages can be difficult to diagnose due to the variable clinical course, although subtle impairments often appear in balance and motor control. The Timed Up and Go (TUG) test is commonly used to assess functional mobility; however, traditional evaluation based solely on total test duration may not be sensitive to early gait alterations. The use of inertial measurement units enables instrumented analysis of individual TUG subphases (iTUG). The aim of this study was determine whether iTUG parameters can help detect balance and movement difficulties indicative of early-stage MS. **Methods**: A total of 30 healthy people and 30 people in the early stages of MS with an expanded disability status score between 1 and 2 were included. The iTUG was performed using three Noraxon inertial sensors placed on the feet and upper spine. **Results**: No significant differences were observed in total iTUG duration between the MS and control groups (*p* = 0.888). In contrast, individuals with MS demonstrated significant differences in spatiotemporal gait parameters, trunk flexion range of motion (*p* = 0.003), number of steps during gait (*p* = 0.004), and turning velocity compared with healthy controls (*p* = 0.008). **Conclusions**: Analysis of iTUG duration is not enough to identify subtle gait and balance impairments in individuals with early-stage MS. Parameters that should be considered when performing an iTUG for the assessment of early stages of MS are spatiotemporal parameters, number of steps, and speed of rotation and subphase times.

## 1. Introduction

Multiple sclerosis (MS) is a chronic, demyelinating inflammatory disease of the central nervous system [1], most common in young adults [2] aged 20–50 years [3]. According to the Multiple Sclerosis International Federation’s’ Atlas of MS 3rd edition report’, the number of people with MS worldwide is estimated to have increased to 2.8 million in 2020 [4], representing a 30% increase in the number of patients compared to 2013 [4,5]. According to Walton et al. [5] the incidence of MS in 2020 was 35.9 per 100,000 people, while data from the Atlas of Multiple Sclerosis indicate that in 2023 approximately 2.9 million people worldwide were living with the disease [6]. The disease affected three times more women than men [7].

Diagnosis of MS includes not only the analysis of clinical symptoms but also imaging studies such as brain and spinal cord MRI and cerebrospinal fluid analysis, which help identify disease biomarkers [8]. However, the misdiagnosis of MS remains a significant clinical problem [9,10], which can lead to delays in treatment and a reduced quality of life for patients. The diagnosis is complicated by the presence of a wide variety of disorders [11], so the disease can present with very different courses [12].

The most common symptoms of MS are visual disturbances [13], sensory disorders [13], motor dysfunctions [14,15], muscle weakness [14,16], and balance problems [14,16]. The research by Ghasemi et al. [14], Hudgens et al. [3], and Kister et al. [13] shows that the disease causes problems with balance and gait, and, most importantly, causes faster fatigue, resulting in a disability [3]. The result is a reduction in activity [17], participation in society [18], or limitations affecting the performance of activities of daily living [13]. This highlights the need for early detection of deterioration in motor and balance functions, which may indicate the progression in early stages of the disease.

One of the standards to determine the stage of the disease is the Expanded Disability Status Score (EDSS) [19]. It is a ten-point scale, with low scores indicating early stages of the disease [19]. It is based on the assessment of visual function, brainstem, pyramidal tract, cerebellum, sensory system, and higher cerebral functions, but also includes an assessment of mobility [20].

Gait and balance disorders are among the most common symptoms of multiple sclerosis and can appear early in the progression of the disease. Even with low EDSSs, patients may exhibit subtle alterations in gait parameters, such as a shortened stride length, increased gait variability, or postural control disorders [21,22]. These alterations are not always identified during routine clinical assessments. The early detection of such abnormalities is important because gait disturbances are associated with an increased risk of falls, a reduced quality of life, and progressive functional disability.

In recent years, objective measurement methods using assistive technologies, such as motion analysis systems and inertial measurement units (IMUs), have become increasingly important in assessing gait function in neurological disorders [23]. These tools enable detailed assessment of temporal-spatial and kinematic gait parameters, identifying changes invisible in standard clinical tests. Using such technologies alongside traditional functional assessment tools can be valuable, particularly for the early detection of gait disorders and monitoring their progression in patients with MS.

In recent years, there has been a growing need for simple and rapid tools to assess motor function that can be used for both diagnosing and monitoring disease progression [23,24,25]. One such tool is the Timed Up and Go (TUG) test [26].

The TUG is a physical fitness test often used by clinicians and physiotherapists [27] because of its association with fall risk [1]. It is not only used in patients with neurological disorders such as Parkinson’s disease [28] or Alzheimer’s disease [29] but also in the elderly population [30] or assessment of changes in functional capacity following COVID-19 infection [31]. It aims to assess functional mobility [12], and, in the case of patients with medical conditions, to monitor disease progression or the effects of treatment [32].

Until now, physiotherapists have analyzed the total duration of the test, but with the development of motion capture systems and inertial sensors, there is a growing trend in the literature to analyze not only the total duration but also each phase of the test, e.g., standing up, walking, turning, and sitting down. For example, the TUG test is increasingly being performed using inertial sensors. In such publications, this test is referred to as the instrumented Timed Up and Go test (iTUG).

The use of IMU sensors during the TUG test opens up more research possibilities, allowing a wider range of variables to be identified for analysis, which can provide valuable new diagnostic information [33,34]. The evaluation is not only based solely on the timing of the test. When performing the iTUG test, it is possible to determine the duration of each phase, such as standing up, walking, turning, and sitting down. In addition, a detailed gait analysis can be performed. This includes the determination of temporal-spatial and kinematic parameters. Furthermore, you can measure various variables, such as turning speed, number of steps taken, and range of motion. The aim of this study was to investigate whether the parameters of the TUG test could help detect imbalance and mobility difficulties that may indicate the early balance and gait disorders, not present in routine clinical assessment of MS patient.

The authors hypothesize that the use of IMU sensors during the iTUG test will allow the determination of additional parameters during the different phases of the test that may differ between MS patients and healthy subjects. It is hypothesized that the parameters of the iTUG test will help detect imbalances and mobility difficulties that may be present in early stages of MS.

## 2. Materials and Methods

In this study, the iTUG test was performed using inertial sensors in people with multiple sclerosis and in a control group.

### 2.1. Participants

The study was cross sectional. Patients from the Neurology Department of the Central University Hospital (Katowice, Poland) who met the inclusion criteria and gave their written consent were included in the study.

The research procedure was approved by the ethical committee of the Medical University of Silesia, Poland (BNW/NWN/0052/KB1/76/23).

Participants were included in the study group if they had a confirmed diagnosis of multiple sclerosis by a neurologist in accordance with McDonald’s criteria [35,36] and an EDSS of 1–2. Participants were excluded if they were unable to walk independently or had previously sustained injuries (e.g., fractures or sprains).

Participants in the control (healthy) group were recruited from the local community on a voluntary basis. The criteria for inclusion in the study were the absence of diagnosed neurological diseases, musculoskeletal disorders, and balance disorders. Exclusion criteria included a history of neurological diseases, lower limb injuries within the last six months, vestibular disorders, use of walking aids, and other conditions that could affect gait or postural stability.

A sample size/power analysis was conducted at G*Power 3.1.9.7. To calculate a representative sample size for the t-student test/Mann–Whitney U test involving two groups, the expected effect size (effect size d = 1.28), significance level (α = 0.05), and statistical power (1 − β = 0.95) were calculated. Appropriate statistical formulas and data from previous studies [37] were used to calculate the minimum number of participants. These calculations aimed to ensure adequate statistical power to detect significant differences between the groups. G*Power indicated a total sample size of 28 participants in two groups (14 participants in one group). It was decided to expand this group to include 30 people with multiple sclerosis.

### 2.2. Instrumented Timed Up and Go

The iTUG test was performed using Noraxon MyoMotion MR4.0.90 inertial sensors (Noraxon U.S.A., Inc., Scottsdale, AZ, USA). Three sensors were used to record motion (sampling rate: 200 Hz), placed according to the manufacturer’s recommendations, on the upper spine (C7 circle) and feet.

**Figure 1:**
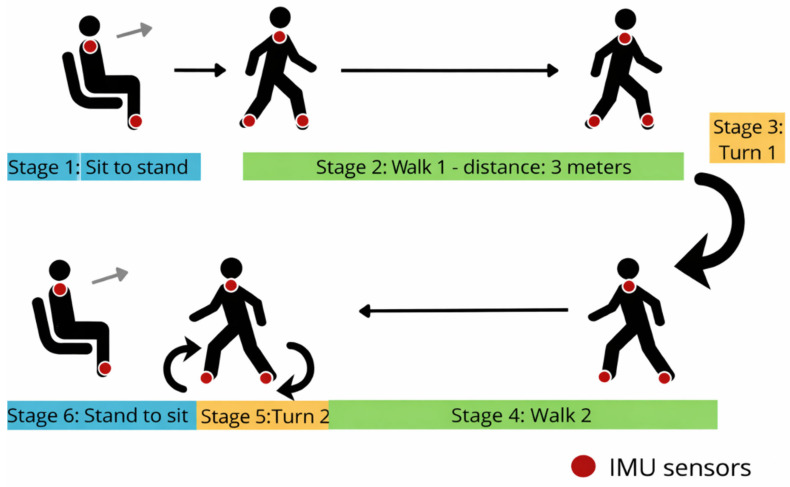
Stages of the Timed Up and Go (TUG) test with placement of IMU sensors on both feet and the upper spine.

The Up and Go test was conducted according to available guidelines [38]. The performance of the Up and Go test consisted of six phases. At the beginning of the test, the subject was asked to stand up from a chair without the help of the upper limbs, then walk a distance of 3 m to a bollard (stage 2), where they would turn (Stage 3) and return to the chair (Stage 4), and then turn again (Stage 5) and return to a sitting position on the chair (Stage 6). The subject was free to choose which lower limb started the movement (Figure 1).

The iTUG test was used to determine the duration of each phase: standing up, rotation 1, rotation 2, gait time (sum of walk 1 and walk 2), and sitting down. In addition, the Noraxon system made it possible to determine the spatiotemporal parameters of movement in the individual test phases (e.g., number of steps) and the angular velocity of trunk rotation in relation to the vertical, transverse, and sagittal axes.

The following parameters were analyzed: duration of the whole test and each of its phases, walk cadence, stride time, step count, trunk flex. ROM, Trunk Lat. ROM, step count, trunk rotation speed maximum, and stride time. All data were determined using the Noraxon MR4 system and are presented in Table 1.

### 2.3. Statistical Analysis

In the present study, statistical analysis of the results obtained was carried out using the Jasp 0.95.4 software. Descriptive statistics were used to characterize the study groups. Quantitative variables were presented as mean ± standard deviation. Additionally, the minimum and maximum values are presented. Statistical analyses were performed at the *p* < 0.05 level of significance. The normality of the distribution was tested using the Shapiro–Wilk test (*p*-value < 0.05 indicates normal distribution). The significance of differences in the quantitative variables analyzed between groups was tested for normality of distribution using Student’s *t*-test or Mann–Whitney U test. The effect size was calculated as Cohen’s d (for the *t*-test) and the rank-sum correlation (for the Mann–Whitney U test).

## 3. Results

### 3.1. Participant Characteristics

The group of people suffering from multiple sclerosis (Group with MS) consisted of 30 patients (19 women and 11 men) with a mean age of 33.83 ± 10.01 and a mean EDSS of 1.58 ± 0.35. The control group consisted of 18 women and 12 men with a mean age of 33.13 ± 11.74 years. Details of the study groups are shown in Table 2.

### 3.2. Results of the Timed Up and Go (TUG) Test Duration

The study assessed the mean times values for the whole test and each phase of the Up and Go test for the MS and control group (Table 3).

The study found that there was no statistically significant difference in the average duration of the whole Up and Go test, with averages ranging in control group from 6.13 s to 10.98 s in MS group and 7.29 s to 12.72 s in control group.

Significant differences between the groups were found for standing up, turn 1, and turn 2. It is worth noting that the differences in gait phase time between the groups are also borderline significant. Higher mean values were obtained for people with MS.

### 3.3. Results of Parameters Derived from the Sit-to-Stand, Walking, and Turning Phases

The determined spatiotemporal parameters for gait, the number of steps during gait phases, trunk range of motion, the number of steps during turn 1, maximum speed of turn 1, and step time during turn 1 for the control group and group with MS are presented in Table 4.

Significant differences were found between the control group and the group with MS for the spatiotemporal parameters, maximum average speed of rotation, stride time in Turn 1, and ranges of motion but only for trunk flexion. No differences were found for two parameters, the number of steps during the first rotation and range of motion for trunk lateral.

Higher cadence and number of steps and lower stride time were recorded in the MS group compared to the control group.

## 4. Discussion

MS could be difficult to diagnose clinically in the early stages of the disease, and its symptoms are variable and unpredictable [39]. One of the most common problems reported by patients is muscle weakness [1,40], which is directly related to the deterioration of motor function [41]. The EDSS is widely used for assessment of the disease progression; however, the scale does not allow objective assessment of the gait and balance. In clinical practice, tests such as the six-minute walk test [21], the stabilography test [22], or the Up and Go test [42] are performed to monitor the progression of the disease. The advantage of the TUG test is its complexity. It covers several key activities of daily living, such as getting up, sitting down, walking, and turning, which are often difficult for people with MS. The use of motion capture systems and IMU sensors during the TUG test expands its diagnostic potential. It provides a comprehensive assessment of both gait and the ability to perform complex movements.

The aim of this study was investigate whether the iTUG test parameters could help detect imbalance and mobility difficulties consistent with early MS stages.

The obtained results suggest that the use of IMU sensors during the iTUG test enables the identification of additional parameters at different stages of the test that may differ between individuals with MS and healthy controls. These results indicate that iTUG parameters may reveal subtle imbalance and mobility difficulties not captured by self-reports or by the EDSS scale.

The study analyzed the performance of people with early MS and young adults using the TUG test, enhanced by the use of three IMU sensors. The study group included people who met the criteria for early stage of MS, defined as an EDSS parameter score between 1 and 2.

The use of inertial sensors during the TUG test allows not only the total duration of the test to be more accurately determined but also the timing of each phase of the test, an assessment of the gait, and an analysis of the rotation phases. These are elements that seem to be able to determine whether the person being tested has a balance or movement disorder. The results suggest that people with early-stage MS can achieve similar total TUG times as healthy controls, but detailed phase analysis reveals subtle differences in movement strategies. The mean time obtained by the group with MS, in the early stages of the disease, was 8.78 ± 1.2 s, which was similar to the result of the control group and was not statistically different. It is noteworthy that a higher mean test time was obtained in the study by Pau et al. [1], 13.92 ± 6.5 s for women with MS and 12.68 ± 3.83 s for male patients. The higher values may have been due to the greater severity of the disease, as the mean EDSS in the Pau et al. [1] study was 3.3 ± 1.9 for women and 3.6 ± 1.3 for men, whereas in our study, the EDSS averaged 1.57 ± 0.38. The higher total test time obtained by Pau et al. [1] was secondary to higher individual phase times. Ciol et al. [43] performed the TUG test in three groups of MS patients (group I EDSS from 0 to 3.5, group II EDSS from 4 to 5.5, and group III EDSS above 6). The shortest time was obtained by group I. The authors of the paper [43] observed an increase in the TUG time with increasing EDSS, and a clear increase was observed, especially between group I and group II [43]. Moon et al. [37] conducted a study in groups with different stages of MS. The group included 15 people with MS with a mean EDSS of 1.5 (ranging from 0 to 2.5) and obtained a shorter mean time of 7.28 ± 1.19 s, results comparable to ours.

From the results of this study, together with findings from Ciol et al. [43] and Moon et al. [37], it can be concluded that people with MS in the early stages of the disease can achieve similar TUG test times as a control group. This may suggest that monitoring only the duration of the whole test may not be sufficient to detect changes in the early disease stage.

While total TUG time did not differ significantly between early-stage MS patients and controls, analysis of individual phases revealed significant differences in movement organization. This phenomenon is consistent with reports in the literature indicating more frequent problems with movement initiation in people with MS [44]. At the same time, a significantly shorter duration of the turning phases was observed compared to the control group, which may suggest compensatory shortening or simplification of movement at moments of change in direction. While the overall test result remained similar, analyzing the individual phases revealed significant differences in movement organization that would otherwise remain invisible. One of the parameters analyzed during gait was stride time, which averaged 0.91 ± 0.1 s in people with MS, representing a shorter stride time of almost 13% compared to the control group. Similar results were observed in a study by Moon et al. [37], in which the mean stride time during gait in the TUG test was 0.929 ± 0.085 s in a group of MS patients with a mean EDSS of 1.5.

The TUG test has the advantage of combining several activities of daily living. In particular, the 3 m gait phase allows the assessment of temporospatial parameters (Table 3). The value of the stride time parameter can also be compared with the results of other studies focusing on gait analysis. In the work of Sosnoff et al. [45], the mean value of stride time in patients with an EDSS in the range of 1–3.5 was 1.08 s, which is about 20% more than the results obtained in the present study [37]. However, it should be emphasized that the participants in Sosnoff’s study had a higher level of disability (EDSS), which may have contributed to the longer step time.

Another parameter that can be assessed during the gait phase of the TUG test is walk cadence and the number of steps taken. The obtained walk cadence was 11 steps/minute higher compared to the results of Berg-Hansen [21], who used a six-minute gait test to evaluate gait parameters in patients with MS. It was noted that the walk cadence and the number of steps taken during the gait phase are higher and statistically significantly different in the MS group compared to the control group, i.e., the cadence during the gait phase was 1.39% higher, and the number of steps during the gait phase was 25.86% higher. These findings may suggest that individuals with MS tend to take shorter, more frequent steps. The observed increase in cadence can be partly explained by the weakness of the lower limb muscles, which is observed in people with multiple sclerosis. Weakness of the lower limb muscles can cause a reduction in stride length and shortening of the stride, leading to a compensatory strategy of shorter, more frequent steps in order to maintain postural stability during walking [46]. Such adaptation may help maintain balance and reduce the risk of falls during functional mobility tasks such as the TUG test. These results highlight the importance of considering muscle activity when interpreting gait parameters, especially in studies using an instrumental version of the TUG test.

The statistically significant differences obtained in all analyzed parameters related to the gait phase suggest that motion analysis using inertial sensors could be used for more than just the iTUG test. This technology could also be implemented in other standardized functional tests involving gait, such as the 10-Meter Walk Test (10 MWT) [47] or the 25-Foot Walk Test (25FW) [48]. Including inertial sensors in these procedures makes it possible to supplement traditional assessments with objective, quantitative data on temporal and spatial gait parameters, postural control, and locomotor strategies, which classic task completion time measurements do not provide. This could enhance the ability of tests to detect subtle functional changes in the early stages of MS and provide valuable support for long-term monitoring of disease progression and evaluation of the effectiveness of therapeutic interventions.

It is worth noting the results obtained from the rotational phase, i.e., number of steps, max trunk rotational speed, and stride time. MS patients showed significantly lower maximum trunk rotational speed and shorter stride time compared to controls. The number of steps during turn phase 1 was higher in the MS group, though this difference was not statistically significant; nevertheless, it may suggest initial imbalance that becomes apparent during rotation.

A study by Adusumili et al. [49] indicated that data from the pivot phase during TUG testing, such as peak pivot velocity, increased the predictive power in assessing a patient’s balance and limitations. These findings are supported by the data obtained in the present study.

Based on the present study and the work of Ciol et al. [43] and Moon et al. [37], it can be seen that there is a significant decline in motor skills during the early and middle stages of the disease. This phenomenon is particularly evident in studies where the range of EDSS values included higher ranges (above EDSS 3)—in such cases, the mean parameter values were significantly higher.

Monitoring only the total duration of the test is insufficient to detect subtle abnormalities related to postural control and movement organization in people in the early stages of multiple sclerosis. While individuals with MS may achieve comparable results to those in the control group, a detailed biomechanical analysis of gait parameters and the times and movements occurring during the swing phase reveals differences in how the task is performed. This suggests that a more comprehensive understanding of the early changes in mobility requires an evaluation of movement quality and structure—including kinematics and motor strategies—rather than merely measuring the time taken to complete the test.

### Limitations and Directions of Further Research

It should be noted that in the literature the TUG is not only performed for 3 m, but also for 7 m [49] and 10 m [50]. It should be considered whether increasing the distance would be beneficial by increasing the stride or the number of cycles, thus basing the time and space parameters on more data. However, increasing the distance may lead to more fatigue in the subjects.

An important direction for future research is determining the iTUG test parameters that differentiate results according to disease stage. Further studies involving larger groups of patients, including those with different EDSSs, are needed to verify whether differences observed in the early stages of the disease become more pronounced and can serve as reliable indicators for the early detection of gait and balance disorders in people with MS. Understanding how test results change with disease progression could improve monitoring and support the selection of personalized therapy, enabling more precise tracking of changes in patients.

## 5. Conclusions

The results of this study indicate that there is no statistically significant difference in the total duration of the TUG test between the control group and the group with early MS. However, significant differences were observed in the stand, turn 1, and turn 2 phases when the duration of the subphases in the TUG were analyzed.

This may indicate the usefulness of these parameters as factors distinguishing control groups from groups in the early stages of MS. This is all the more so because the scientific literature indicates that the time result of this test alone may be used to monitor the progression of MS.

Analysis of rotation and temporal and spatial gait parameters, such as number of steps, and rotation speed using the iTUG test showed significant differences between the group with MS and the control group. These results suggest that a detailed assessment of these parameters may be useful in identifying early imbalances and locomotor function in patients with MS.

The use of IMU sensors in the TUG test can be an additional tool supporting the work of a neurologist, but it should not replace the methods used so far. It allows for the analysis of a larger number of parameters related to mobility and gait, which enables quantitative assessment and facilitates the monitoring of disease progression in subsequent stages of treatment.

It should be noted that this study included only a small number of participants in the early stages of the disease (EDSS 1–2), which may be a limitation of the study. Further studies involving larger groups of patients at different stages of the disease are needed to confirm whether these correlations are useful for the early detection of gait and balance disorders.

## Figures and Tables

**Table 1:** Description of the parameters determined from the TUG test (ROM—range of motion, deg—degree, s—seconds, flex—flexion, lat—lateral).

Stages	Parameter	Unit	Description
Stages 1–6	Total time	[s]	The total time required to complete the test.
Stage 1	Stand Duration	[s]	The time required to move from a sitting to a standing position.
Stage 2 and 4	Walk Duration	[s]	The time needed to cover a distance of three meters twice.
Stage 3	Turn 1 Duration	[s]	Time required for rotation number 1.
Stage 5	Turn 2 Duration	[s]	Time required for rotation number 2.
Stage 6	Sit Duration	[s]	The time required to move from a standing to a sitting position.
Stage 2 and 4	Walk Cadence	[steps/min]	Number of steps per minute.
Stage 2 and 4	Stride Time	[s]	The time required to complete one step.
Stage 2 and 4	Step Count	[steps]	Number of steps taken in walk 1 and walk 2
Stage 1	Trunk Flex. ROM	[deg]	Range of motion of trunk flexion
Stage 1	Trunk Lat. ROM	[deg]	Range of motion of lateral trunk movements
Stage 3	Step Count	[steps]	Number of steps taken during rotation
Stage 3	Trunk Rotation Speed Max	[deg/s]	Maximum torso rotation speed during the first rotation.
Stage 3	Stride Time	[s]	The time required to complete one step during the rotation phase.

**Table 2:** Characteristics of study participants.

	Group with MS	Control Group
Number of participants	30 (19 Female/11 Male)	30 (18 Female/12 Male)
Age (mean ± SD), years	33.83 ± 10.01	30.13 ± 11.74
Height [m]	1.70 ± 0.07	1.71 ± 0.09
Duration of disease [years]	7.70 ± 7.55	NA
EDSS	1.58 ± 0.35	NA

**Table 3:** Comparison of average times for the whole test and individual phases (* *p* < 0.05, Student’s test or Mann–Whitney U test, [s]—second, flex—flexion, lat—lateral).

Variable	Group with MS			Control Group			*p*	Cohen’s d or the Rank-Sum Correlation
	Mean ± SD	Min	Max	Mean ± SD	Min	Max		
Total time [s]	8.78 ± 1.2	7.29	12.72	8.7 ± 1.18	6.13	10.98	0.888	−0.022
Stand Duration [s]	1.38 ± 0.17	1.15	1.88	1.21 ± 0.22	0.73	1.61	0.005 *	0.419
Walk Duration [s]	3.43 ± 0.93	2.1	6.7	2.99 ± 0.85	1.75	5.34	0.066	0.278
Turn 1 Duration [s]	1.62 ± 0.18	1.29	2.07	1.9 ± 0.29	1.34	2.33	<0.001 *	1.184
Turn 2 Duration [s]	0.89 ± 0.29	0.48	1.57	1.13 ± 0.16	0.8	1.5	<0.001 *	1.005
Sit Duration [s]	1.46 ± 0.23	1.06	1.92	1.47 ± 0.3	0.91	1.98	0.852	0.049

**Table 4:** Comparison of means values of temporal and spatial parameters, ranges of motion, number of steps, and speed of rotation (* *p* < 0.05 Student’s test or Mann–Whitney U Test, ROM—range of motion, deg—degrees, [s]—second, flex—flexion, lat—lateral).

Stage	Parameter	Group with MS			Control Group			*p*	Cohen’s d or the Rank-Sum Correlation
		Mean ± SD	Min	Max	Mean ± SD	Min	Max		
Stage 2 and 4	Walk Cadence [steps/min]	132.01 ± 13.24	96.43	157.07	117.44 ± 12.77	99.45	151.9	<0.001 *	−1.12
Stage 2 and 4	Stride Time [s]	0.91 ± 0.1	0.75	1.25	1.03 ± 0.1	0.86	1.22	<0.001 *	−0.586
Stage 2 and 4	Step Count [steps]	5.5 ± 1.59	3	9	4.37 ± 1.47	2	8	0.004 *	0.424
Stage 1	Trunk Flex. ROM [deg]	25.84 ± 8.56	11.27	52.59	34.38 ± 12.9	15.76	62.59	0.003 *	−0.454
Stage 1	Trunk Lat. ROM [deg]	7.67 ± 4.88	1.19	24.19	11.11 ± 9.85	2.54	44.05	0.432	−0.123
Stage 3	Step Count [steps]	2.7 ± 0.75	2	4	2.23 ± 0.68	1	3	0.054	0.29
Stage 3	Trunk Rotation Speed Max [deg/s]	97.14 ± 36.11	41.84	193.38	70.78 ± 39.56	20.94	172.12	0.008 *	0.396
Stage 3	Stride Time [s]	0.9 ± 0.17	0.58	1.4	1.04 ± 0.14	0.75	1.24	0.003 *	0.846

## Data Availability

The raw data supporting the conclusions of this article will be made available by the authors on request.

## Abbreviations

The following abbreviations are used in this manuscript:

MS	Multiple sclerosis
TUG	The Timed Up and Go test
iTUG	Instrumented Timed Up and Go test
